# The observation of supercapacitor effects on PEMFC–supercapacitor hybridization performance through voltage degradation and electrochemical processes

**DOI:** 10.1039/d0ra00468e

**Published:** 2020-04-01

**Authors:** Rungsima Yeetsorn, Yaowaret Maiket, Wattana Kaewmanee

**Affiliations:** Department of Industrial Chemistry, Faculty of Applied Science, King Mongkut's University of Technology North Bangkok Bangkok 10800 Thailand rungsima.y@sci.kmutnb.ac.th; Thai-French Innovation Institute, King Mongkut's University of Technology North Bangkok Bangkok 10800 Thailand

## Abstract

In the cities in the future, seeing electric vehicles on the roads will be as ordinary an occurrence as seeing internal combustion engine cars today. Electric vehicles can greatly benefit from utilizing polymer electrolyte membrane fuel cells (PEMFCs) because they provide higher efficiency (40–50%) and are more environmentally friendly. However, there are some major drawbacks to using PEMFCs as electrical sources in vehicles; these are energy balance and management issues that must be addressed to meet vehicle power and energy requirements. Therefore, it seems that hybridizing PEMFCs with energy storage devices, such as supercapacitors (SCs), would be an efficient solution to address these drawbacks in order to accommodate driving behaviors such as dynamic loads. The goal of this research is, therefore, to demonstrate the use of a PEMFC–SC direct hybridization configuration with a dynamic stress test by simulating driving behavior in urban areas such as Bangkok. This research presents substantial advantages in energy management and voltage and material degradation. In order to achieve this objective, a quasi-static stress profile, including stationary conditions, load variations, and start–stop conditions, was specifically created for PEMFC–SC direct hybridization systems with 840 hours of operating duration. The performance, durability, and reliability of this system were investigated *via* polarization curves, hysteresis loops, and voltage degradation rates. Then, experimental results were compared to the degradation of the cell components. Any degradation in material components was observed through electrochemical impedance spectroscopy (EIS) and morphology studies. The characterization of materials in the PEMFC–SC direct hybridization systems *via* chemical and electrochemical analyses is an important approach in material invention and modification for the new generation of PEMFCs. This work strives to pave the way for PEMFC hybridization development to achieve effective commercialization.

## Abstract 2

The in-house fuel cell test station.

## Graphical abstract

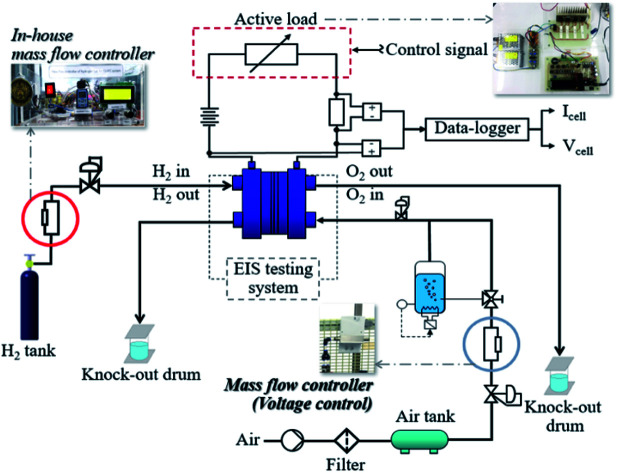

## 1 Introduction

The hydrogen fuel cell technology for new-generation vehicles has received extensive attention as one of the ways to address energy density and autonomy while also not being subject to fast-charging issues. A great example of the acceptance of PEMFCs by the automotive industry is the utilization of PEMFCs as electricity sources in recent years in cars such as the Toyota Mirai, Hyundai HDC-6 Neptune, Mercedes-Benz F-cell, and Honda Clarity. However, the transient response, cost, and weight of these PEMFC systems create imperative challenges that can be addressed *via* vehicle hybridization systems. The power density of PEMFCs in the transient regime remains restricted by the factual response time of the electrochemical reactions. As can be seen, PEMFC is not a suitable solution for the current fluctuations and voltage profiles that occur during vehicle driving. Moreover, peak load demand will promote transient fuel starvation, resulting in irreversible degradation of voltage and material components. The concerning issues of utilizing PEMFCs in transportation may be divided into three main categories as follows: filling fuel, lifespan, and responsiveness.^1^ The lifespan of a PEMFC highly depends on driving behaviors, which are directly related to the operating conditions and the degradation phenomena occurring in the system. For example, the start-up and shut-down cycles as complementary accelerating stress factors will change significantly relative to humidity, cell temperature, and cell pressure; therefore, the fluctuating conditions bring about a reduction in the PEMFC lifespan. In a high-voltage-demand scenario, an overvoltage situation occurs when the cell voltage reaches 1.50 V, which is much higher than the theoretical limit of 1.23 V. The possible causes of overvoltage occurrence are corrosion of the carbon support and platinum (Pt) dissolution,^2^ which are evidence of catalyst degradation. In order to address these issues and facilitate the function of PEMFC systems subjected to dynamic stresses, combining a PEMFC with a secondary power source (battery or supercapacitor)^3^ may be an optimal solution. By working in tandem, this solution enables the PEMFC to slow regime transients by managing high power demand through the storage device. In addition, this type of hybridization provides advantages for fuel cell power downsizing, fuel efficiency enhancement, and regenerative braking energy absorption while also providing a more flexible operating strategy and overcoming fuel cell cold start and transient shortfalls, thus potentially lowering the power cost per unit.^4^ Current hybridization systems have several types of power source combinations, such as fuel cells and backup power, fuel cells and alternative sources, and fuel cells and energy storage systems.^5^ Lithium-ion batteries and supercapacitors are popularly used as energy storage systems in PEMFC hybridization.^6,7^ Lithium-ion batteries show lower power output but higher energy storage capabilities, while supercapacitors provide higher power output and faster charging and discharging. Supercapacitors, on the other hand, can provide transient power to meet load demand quickly; however, they provide relatively low energy-storage capability.^4^ Subsequently, supercapacitors are more suitable to be used as auxiliary sources associated with a main power source, such as a PEMFC. PEMFCs are selected as the main power source because they offer higher energy storage and can supply voltage to the hybridization system for a long term. The working characteristics and efficiency of PEMFC hybridization systems regarding load demands are based upon a powertrain configuration. The powertrain configuration is designed to either directly connect supercapacitors with PEMFCs or to connect supercapacitors with PEMFCs through converters. For the purpose of this research, the direct connecting structure was selected due to the major role supercapacitors play in self-energy management by controlling cell voltage as well as functioning as power generators. The desired function of the supercapacitor is to provide a direct hybridization system and direct protection against rapid power variations, which leads to an increase in the dynamics of the hybridization system. On the other hand, when the supercapacitor in the hybridization is connected to a supercapacitor *via* a converter, it only partially supplies the power needed to balance the voltage level and meet the load demand. Accordingly, this type of PEMFC–SC hybridization cannot respond to the needs of dynamic operation or meet load demand. It is also worth noting that the supercapacitor in the PEMFC–SC direct hybridization system must be pre-charged before utilization to limit the inrush current.^8^ Although several research topics on PEMFC–SC hybridization have received attention from researchers, such as working behavior simulation,^9^ the number of supercapacitors used in the system,^10^ and the powertrain configuration,^11^ the relationships between voltage degradation and material degradation occurring in PEMFC–supercapacitor hybridization systems have not yet been clarified. The degradation mechanisms can be compiled into a database for improvement of cell component performance and durability, hopefully leading to the creation of new materials and new fuel cell hybridization designs in the future. Parameters such as voltage degradation rate,^12^ power, energy, yield, and efficiency, are necessary to determine for consideration of the system performance.^13^ These parameters can be determined based on the following equations:1
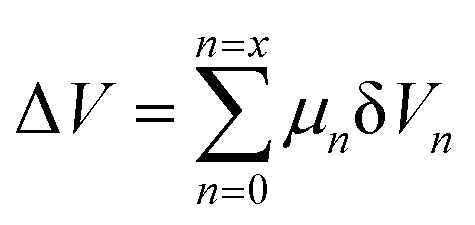
2
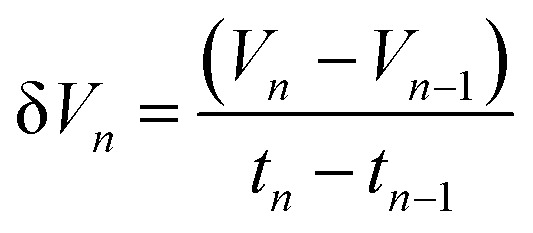
3
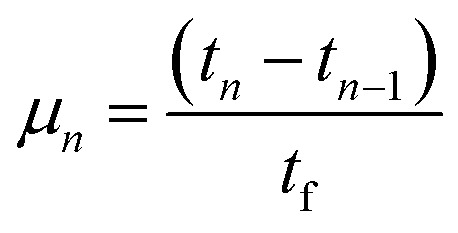

4 *P* = *V* × *I*

5 *E* = *P* × *t*

6
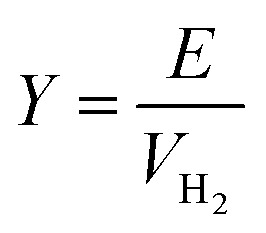
7
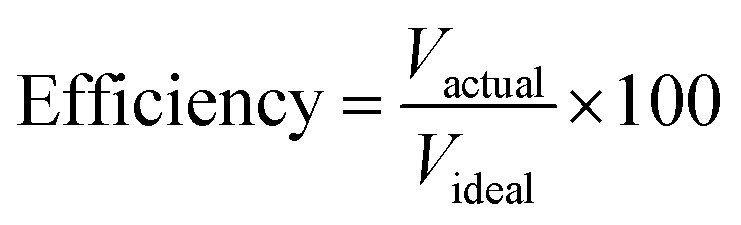
where Δ*V* is the voltage degradation rate (mV h^−1^), δ*V*_*n*_ is the rate of difference between the cell voltage (in mV) at two local time spots *t*_*n*_ and *t*_*n*−1_, and *t*_f_ is the number of time spots used in the test. *P* is power (W), *V* is voltage (V), *I* is current (A), *E* is energy (W h), *t* is time (h), *Y* is yield (W h ml^−1^), and *V*_H_2__ is the hydrogen consumed during production (ml).

Numerous operating conditions can cause degradation of cell components, which are typically generated by electrochemical, thermal and mechanical stresses. There are several types of material degradations, such as corrosion and mechanical failure of bipolar plates or the gas diffusion layer, catalyst corrosion, catalyst dissolution, catalyst surface area reduction, membrane thinning, hot spots on the membrane, and membrane cracking.^14,15^ In automotive applications, a high operating temperature of the PEMFC causes oxidation of the platinum (Pt) catalyst, leading to Pt dissolution and migration.^14^ The resulting changes from the Pt dissolution and migration are Pt band formation and Pt agglomeration, leading to Pt re-deposition/Ostwald ripening, Pt particle growth, Pt surface area reduction, and electrochemical surface area (ECSA) losses.^14,15^ Moreover, the influence of high operating temperatures on the degradation of the polymer structure in the membrane results in peroxide formation. The polymer degradation also generates membrane thinning, pinholes, or membrane cracking; this may result in many complex problems, such as crossover of hydrogen, oxygen, and/or water, hydrofluoric acid and ion release, poisoning, and cation contamination.^14,15^ The operating conditions based on dynamic driving behavior cause reversible and irreversible changes to cell voltage, which also results in water flooding, starvation, *etc.*^16^ The degradation of a PEMFC–supercapacitor hybridization system can be investigated using electrochemical techniques, physicochemical characterization, and morphology observation. The preferable electrochemical techniques used in diagnosing the degradation of fuel cell materials are electrochemical impedance spectroscopy (EIS) and cyclic voltammetry (CV).^17^ EIS can analyze the changes in fuel cell characteristics in terms of electrode–electrolyte interface behavior, double-layer capacity, metal corrosion, electrodeposition, and the electrical properties of the material and interfaces.^18,19^

According to the aforementioned literature, operating PEMFC under sudden load variations, start–stop repetition, or full power conditions will result in performance restrictions on energy balance and in material degradation. The PEMFC must supply uninterruptible and reliable power with constant voltage; otherwise, fluctuating operating conditions will be created. Under the various load demands or various electricity generation levels, the electrochemical reactions occurring in the PEMFC are accelerated. When the redox reactions occur more rapidly, the system will experience higher heat accumulation and lower humidity. These severe circumstances lead to a side reaction in the PEMFC, causing degradation of the fuel cell components. A supercapacitor that achieves fast charge/discharge can balance the energy of the PEMFC with lower accelerated stress on an electrochemical reaction. This implies that the supercapacitor is able to reduce fluctuating operating conditions and material degradation in PEMFC. Created stress test profiles relating to the real dynamic load demand are imperative tools for investigating the feasibility of using supercapacitors for energy balance in a PEMFC–SC hybridization system; meanwhile, they can also be applied to study the effects of this energy management strategy on the degradation of fuel cell materials. Without accelerated stress tests, the study of material degradation phenomena is expensive and requires prolonged testing periods. This research aims to demonstrate the benefit of using a PEMFC and a supercapacitor to create direct PEMFC–SC hybridization and achieve better balance of energy management in addition to the fuel economy of PEMFC–SC direct hybridization. The system performance was validated *via* an accelerated stress test procedure directly based on the power demand in real applications for predicting the causes of degradation of the materials existing in the hybridization system. The experimental results from the accelerated stress tests were represented by voltage production, volume and cost of hydrogen utilization, power density, efficiency, and voltage degradation. EIS, SEM and optical microscopy studies were used to carefully analyze the characteristics of the materials upon completion of cell operations.

## 2 Experimental methodologies

### The in-house PEMFC test station

**Fig. 1:**
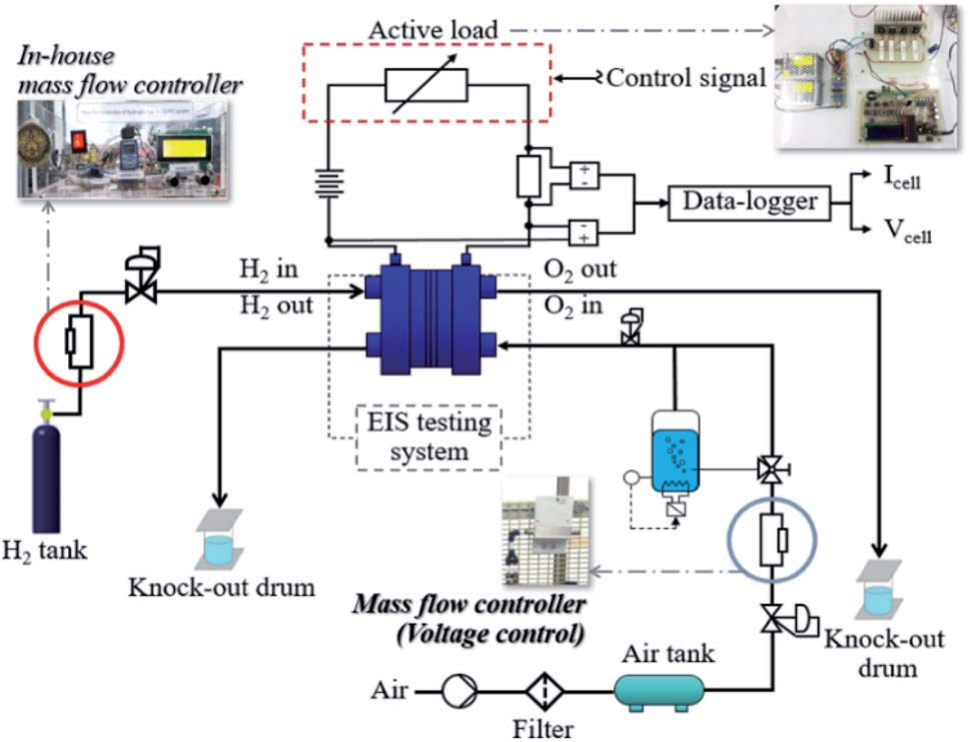
The in-house fuel cell test station.

The test station utilized for the research activities was designed and developed by researchers at King Mongkut's University of Technology North Bangkok. Fig. 1 presents a schematic of the PEMFC benchmark setup. The PEMFC test station was designed to function in different operating modes under different conditions: operating temperature; gas utilization ratio; humidification; rates for inlet hydrogen and air; backpressure; gas temperature; and electric load. The factors under investigation were current, cell voltage, pressure, and temperature during the inlet and outlet gas flow; these were controlled and observed through interface controllers which were also developed using the MPLAB X IDE program and a DL2200/220VAC data-logger (Wisco, Thailand). The electrical connections, gas flows, humidification and cooling systems, and different sensor locations can be found in the schematic in Fig. 1. A mass flow controller with a programmed flow rate was designed and manufactured to ensure that the PEMFC operation was in accordance with the flow rate programming. A working concept of the developed mass flow controller used electrical voltage supplied from a power supply to control the gas valves and change the gas flow rates as programmed.

### PEMFC and supercapacitor specifications

Two F107 PEMFCs (Fuel Cell Store, USA) with an active area of 16 cm^2^ were used for this project. PEMFCs with active areas of 16 cm^2^ are widely used for research on a laboratory scale. These single cells offered maximum performance as follows: 450 mA of current; 0.4 V of voltage; and 180 mW of power. The cells were operated using air and pure hydrogen with a liquid cooling system. The single cells were assembled with perforated metal plates, platinum-coated carbon paper on both the anode and cathode sides, and Nafion 212. The supercapacitor (IOXUS, USA) was incorporated in testing. It was designated with specifications of 2.7 V, 100 F, and 0.10 W h. The selection of the supercapacitor size is relevant to its voltage acceptance ability during the charging process. As mentioned in the introduction section, the connection between the PEMFC and supercapacitor in our hybrid system is a direct connection; therefore, the voltage of the supercapacitor must be higher than the voltage of the PEMFC. This is necessary to prevent current from flowing back from the PEMFC to the supercapacitor. If the supercapacitor experiences overcharging, it may explode. The selected supercapacitor had an electrical potential of 2.7 V, which is higher than the potential of the PEMFC (1.23 V). The selected supercapacitor with specifications of 2.7 V and 100 F is the most popular model and is easily found in Bangkok. Note: an operating temperature of 30 °C with 1 atm of pressure and 100% relative humidity (at 30 °C) were controlled during the period of cell operation. The important operating parameters, namely stoichiometry between the fuel and oxidant, temperature, pressure, and humidity, were set based on thermodynamic efficiency. The optimum stoichiometry of the fuel and oxidant should be in the range of 1.2 to 1.5 to provide excess reactants for driving the redox reaction. The relative humidity is typically controlled at around 98–100% because it significantly affects proton transfer due to the Grotthuss proton transfer mechanism.^20,21^

### The powertrain configuration

**Fig. 2:**
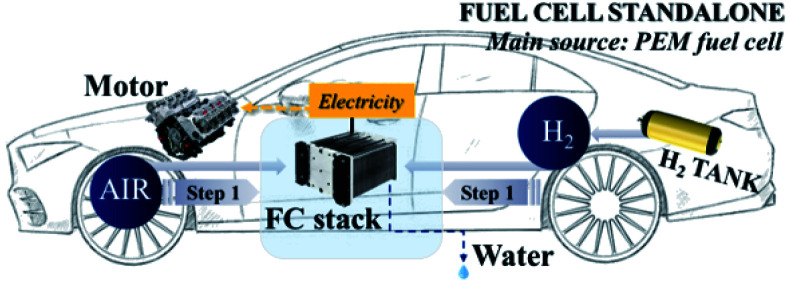
The single PEMFC powertrain configuration.

**Fig. 3:**
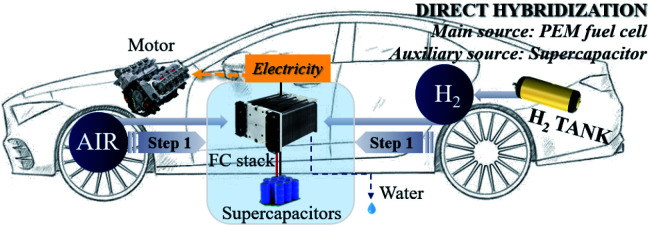
The PEMFC–SC direct hybridization powertrain configuration.

Two powertrain configurations (PEMFC and PEMFC–SC direct hybridization) were utilized to investigate the effects of the supercapacitor on the performance of the PEMFC–SC hybridization system (Fig. 2 and 3). In the powertrain configuration, PEMFC was utilized as a main DC-link voltage to meet the load demand. The output current generated from a PEMFC is limited by the fuel and oxidant flow rate. In the case of PEMFC–SC direct hybridization, a PEMFC was directly hybridized with a single supercapacitor in parallel with two 0.10 m long electric wires. The PEMFC operated as the main power source generator in order to meet load demand, while the functions of the supercapacitor were the charging and discharging processes. The supercapacitor also stored excess power produced from the PEMFC as well as the regenerative braking energy. It acted as a power source, providing power to the PEMFC and meeting the load demand when it contained excess energy. The supercapacitor functioned automatically when the voltage levels of the PEMFC and the load demand were different.

### The stress test profile

**Fig. 4:**
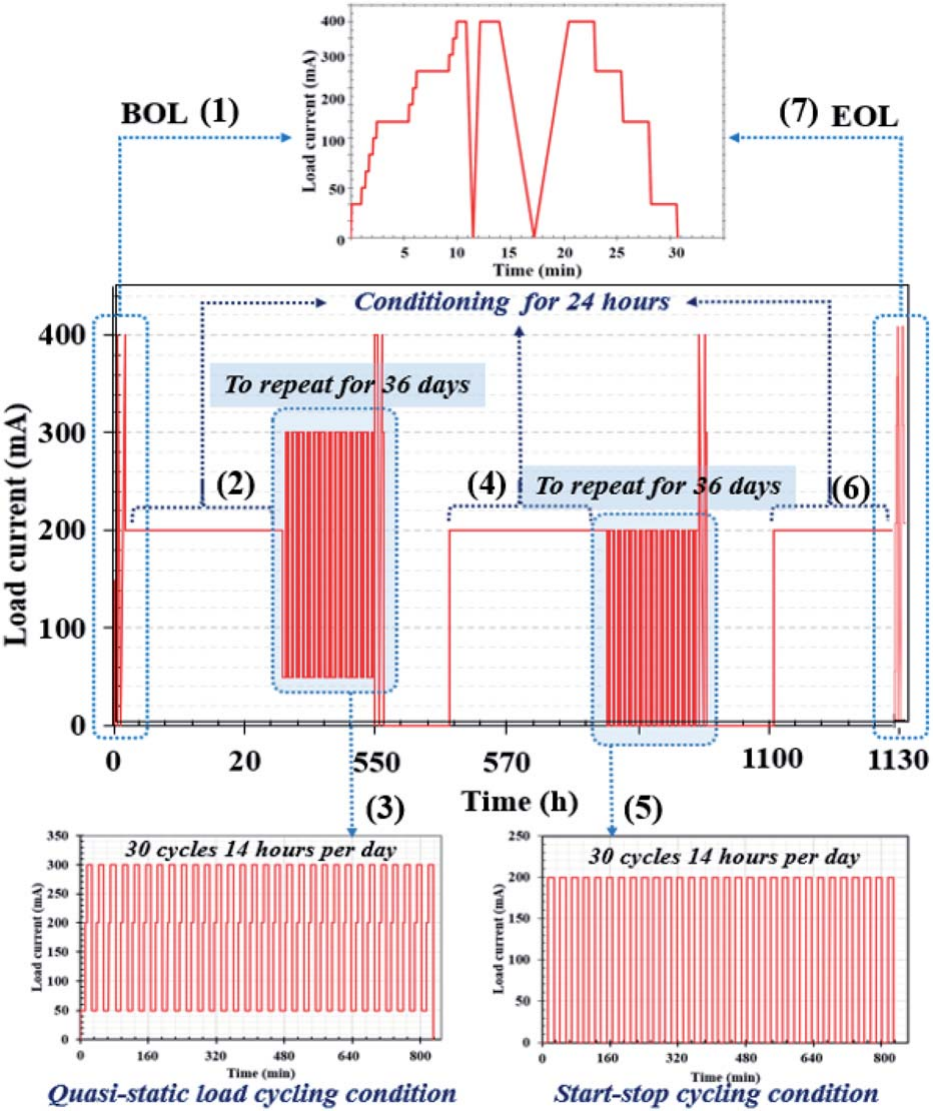
Designed accelerated stress profile based on driving behavior in an urban area. Note: in terms of electrochemical characterization *via* EIS, the acquired data were fitted using the MATLAB program to generate Nyquist plots of the impedance spectra.

The PEMFC and PEMFC–SC direct hybridization systems ran under the maintained operating conditions mentioned above. Two aging modes were used at this stage, load cycling and start–stop cycling, which were applied to the profile procedure. A quasi-static load cycle profile for the hybrid configuration was also considered in this research. The interval in the profile corresponded to normal vehicle utilization; an example of this is daily trips to and from work, as this would also simulate the use of vehicles for other activities in the evening. The accelerated stress profile (Fig. 4) was designed to fit into static conditions, load cycling, and start–stop conditions to find aging correlations and verify the hypothesis. The created profile comprised seven steps, each of which are explained below and in Fig. 4. The performance was validated by polarization curves and the electrochemical impedance spectra. Identical validation procedures were carried out at the beginning and end of the life of the PEMFC, with the validation window scheduled every 24 h.

#### The first step (Fig. 4 (1) at 0 to 25 h)

Preconditioning and system warm-up methods were completed 24 h prior to the beginning of life (BOL) measurements; then, the PEMFC or PEMFC–SC direct hybridization system was constantly operated at 200 mA for 24 h (according to the average generated current). When performing the BOL measurements, loads were gradually applied to generate currents ranging from 50 mA to 400 mA (high current value) at an interval of 50 mA. Note: the maximum electrical current generated by the PEMFC was 450 mA. In Fig. 4, the BOL measurements are imposed to monitor the starting performance *via* polarization curves, as schematized in BOL (1). Moreover, this operating stage involved preconditioning and single cell warm-up.

#### The second step (Fig. 4 (2) at 25 to 49 h)

For the purpose of conditioning and recovering, the PEMFC or PEMFC–SC direct hybridization system operation was maintained constantly at 200 mA. The conditioning period was set at 24 h. Due to the possibility of water flooding during the continuous run, the voltage was recovered by purging the cell with nitrogen gas.

#### The third step (Fig. 4 (3) at 49 to 553 h)

Quasi-static load cycling was operated from 50 mA, which is a minimum constant-current operating value, all the way to the highest current value of 300 mA. The PEMFC or PEMFC–SC direct hybridization system was subsequently operated at 200 mA, in accordance with the average power. A sequence of 30 repetitions within 14 hours was imposed as the load cycles, as schematized in Fig. 4. The duration of each load cycling measurement was scheduled for 504 hours. This step represented the scenario of driving behavior with accelerated speed. It can be referred to as cyclic temperature and relative humidity-generated voltage degradation. The concept of quasi-static load cycling design was motivated by actual driving behavior. In real life conditions, driving speeds cannot be changed immediately because it takes time to gradually accelerate an engine to achieve a desired speed as well as to slow the driving speed.

#### The fourth step (Fig. 4 (4) at 553 to 577 h)

The PEMFC or PEMFC–SC direct hybridization system operation was maintained constantly at 200 mA for conditioning and recovering, as elucidated in the second step.

#### The fifth step (Fig. 4 (5) at 577 to 1081 h)

Hydrogen gas and air zero were not supplied when the start–stop current was cut off. There was no current generated at the stop stage, while the cell was started at 200 mA. The PEMFC system was naturally cooled for 30 minutes when the reactants were cut off. During the cooling period, problems of water saturation and flooding were evaluated before restarting the PEMFC system. If the problems manifested during the cooling period in the PEMFC system, a certain volume of nitrogen gas was injected to purge water from the system. The start–stop cycling test required 504 hours to finish operation, with 30 repetitions within 14 h. The start–stop cycling was designed to observe ageing of the system due to sudden and repeated load variation conditions.

#### The sixth step (Fig. 4 (6) at 1081 to 1105 h)

During this stage, the system was conditioned again.

#### The seventh step (Fig. 4 (7) at 1105 to 1135 h)

The end of life (EOL) step was executed; the measurement was identical to the BOL step.

## 3 Results and discussion

### The influence of the supercapacitor on the performance of the single PEMFC–SC direct hybridization system

#### The importance of pre-charging of a supercapacitor regarding energy management

**Fig. 5:**
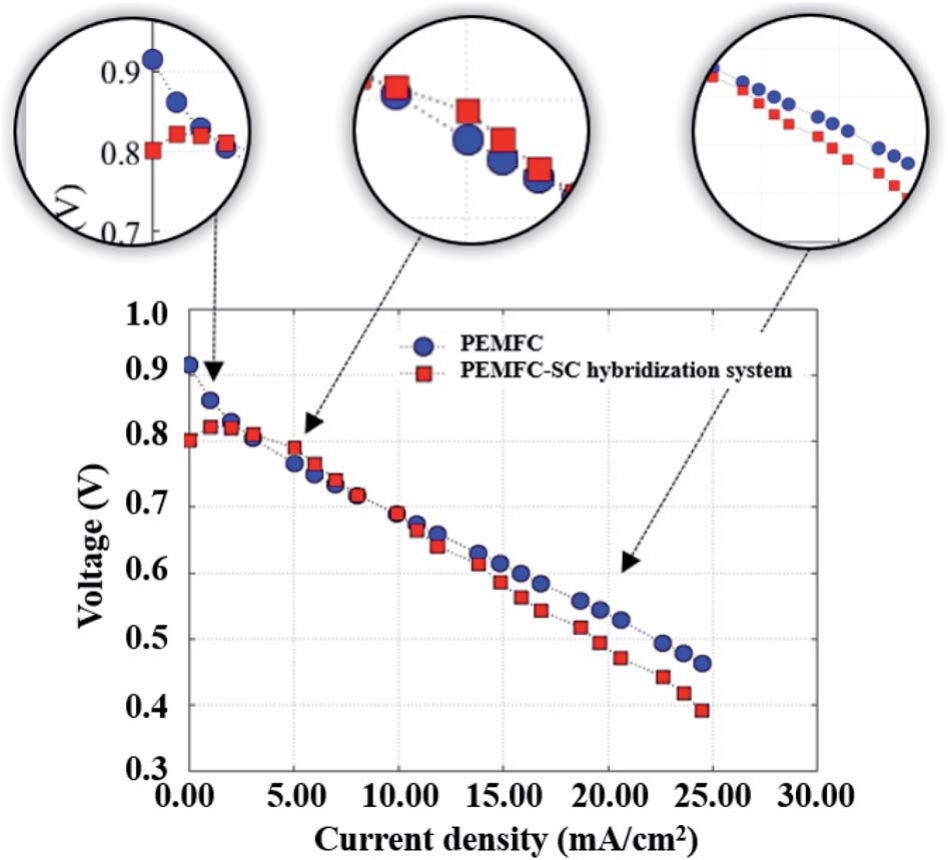
The polarization curves of the PEMFC and PEMFC–SC direct hybridization systems.

A PEMFC continuously delivers energy as long as its fuel and oxidant supplies are maintained. A well-known technical restriction of PEMFCs is that they provide low efficiency in an environment where there is low load demand and a slow power transfer rate in transitory circumstances. Therefore, supercapacitors are introduced to assist PEMFCs in generating power to meet load demands, principally during startup of the system and in transient incidences. The PEMFC–SC direct hybridization setup leads to a decrease in hydrogen consumption. This issue will be described in more detail in the next section of this research. Because a supercapacitor enables fast charging and discharging, it was applied as an auxiliary source in the hybridization system. It is suitable for a load profile containing rapid load changing, such as quasi-static load cycling conditions. According to the accelerated stress profile illustrated in Fig. 4, the hybrid system must generate electricity from 50 mA to 200 mA with an increasing rate of 25 mA min^−1^. If the PEMFC is a standalone electricity generator, fuel and/or oxidant starvation phenomena may occur. These phenomena can exist in the cell starting situation during start–stop cycling as well. In a period of 2 to 3 minutes after the reactants are supplied during start–stop cycling, the PEMFC will still generate electricity due to the remaining reactant. Following that, the system gains excess energy, caused by the electrolysis process. The electrolysis produces a mix of hydrogen and oxygen gases that is the source of voltage degradation. If a supercapacitor is present in the system, the excess electrical energy will be transferred to it; therefore, the materials will be protected from degradation. The polarization curves of the standalone PEMFC and PEMFC–SC direct hybridization systems were measured and are displayed in Fig. 5.

A single PEMFC generated 0.90 V of cell voltage under open circuit voltage (OCV) conditions, whereas the PEMFC–SC direct hybridization system produced around 0.80 V of cell voltage. This was an unexpected result because a supercapacitor should enhance the electrical energy production of a system. The reason that the PEMFC–SC direct hybridization system delivered lower cell voltage is related to the charging process of the supercapacitor from the PEMFC. At the beginning, the cell voltages of the PEMFC and supercapacitor were balanced in the medium current density range. Following that, the supercapacitor was charged again with a higher current density; following that, the cell voltage drop of the PEMFC–SC direct hybridization system was higher than the voltage drop in the standalone PEMFC. These results suggested the idea to pre-charge the supercapacitor before connecting it with the PEMFC.

**Fig. 6:**
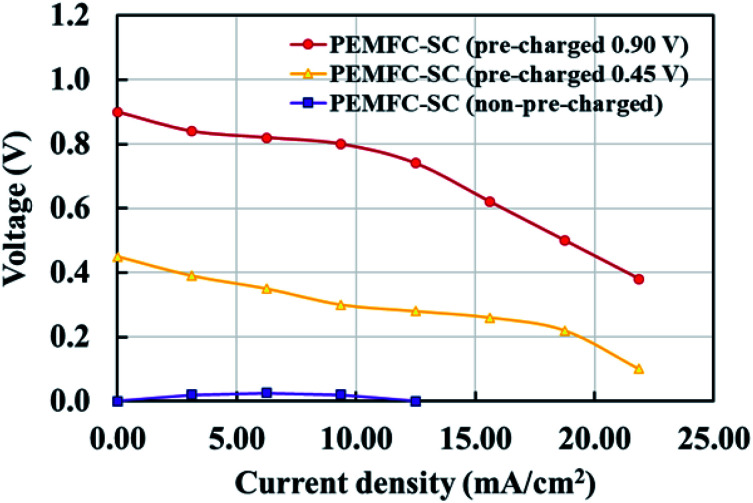
The effects of pre-charging the supercapacitor on the performance of PEMFC–SC direct hybridization systems.

**Fig. 7:**
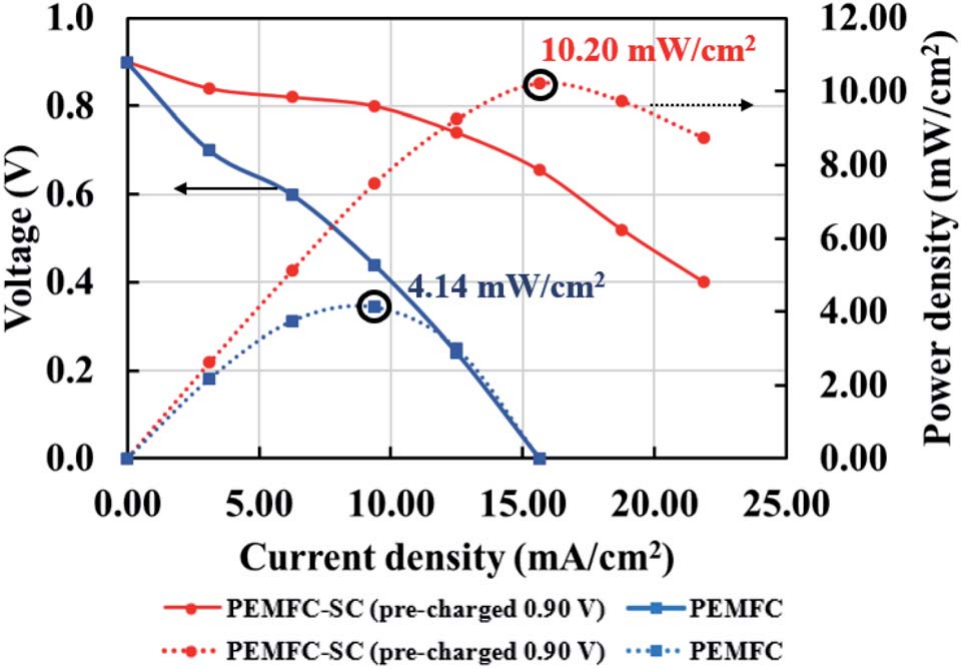
Polarization and power density curves of the PEMFC and PEMFC–SC direct hybridization systems.

Fig. 6 illustrates the effects of pre-charging on system performance. The supercapacitor was pre-charged with voltages of 0.90 and 0.45 V. When selecting the supercapacitor charging level, it is important to note that it should not exceed the power generation capability of the PEMFC. If the supercapacitor is charged to a higher electrical potential than the PEMFC, the electrical energy may transfer to the PEMFC, resulting in electrolysis or deterioration of the materials inside the PEMFC. The results showed that pre-charging the supercapacitor improved the overall system performance by conserving cell voltage levels, leading to decreased losses in all current density regimes. The hybridization system connected with the 0.90 V pre-charging supercapacitor offered 2.46 times higher power density than the system connected with the non-pre-charging supercapacitor (Fig. 7). Note: the 2.46 times higher power density was determined by dividing the power density of the PEMFC–SC hybridization system (10.20 mW cm^−2^) by the power density of the PEMFC (4.14 mW cm^−2^).

#### Fuel economy analysis of the PEMFC and PEMFC–SC direct hybridization systems

While the PEMFC has higher energy density,^19^ it cannot provide stable electrical power under dynamic load conditions; a supercapacitor, on the other hand, is much more stable under load fluctuations. Fuel economy is another issue of concern when modifying PEMFC–SCs for direct hybridization.

The current created from the PEMFC will increase with the hydrogen flow rate according to Faraday's law.^22^ To calculate the cost of hydrogen for generating 1 mA of electricity, the hydrogen flow rate at 200 ml min^−1^ was controlled from the stoichiometry criteria (eqn (8)). According to a theoretical calculation, a hydrogen flow rate of 200 ml min^−1^ is required to generate maximum current and voltage from the F107 PEMFC. This can guarantee that the quantity of hydrogen will be enough for fuel cell operation even if the load demand is fluctuating.8
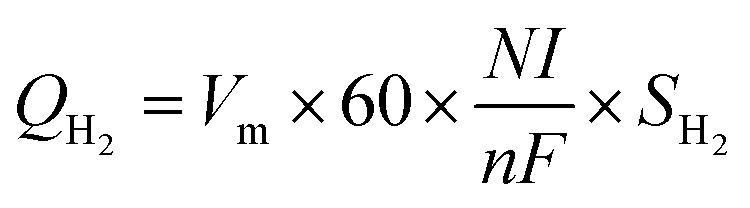
where *Q*_H_2__ is the hydrogen flow rate (ml min^−1^), *V*_m_ is the molar volume (*V*_m_ = *RT*/*P*), *R* is the gas constant (0.082 L atm mol^−1^ K^−1^), *T* is the temperature (K), *P* is the pressure (atm), *N* is the number of cells, *I* is the current (A), *n* is the number of electrons transferred (mol), *F* is Faraday's constant (96 485 C), and *S*_H_2__ is the stoichiometry ratio of H_2_ (eqn (9)); *S* = 1 indicates enough gas molecules are provided, *S* > 1 indicates excess gas molecules are provided, and *S* < 1 indicates insufficient gas molecules are provided.9
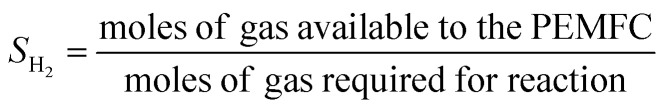


The results in Table 1 indicate that the supercapacitor did not support the PEMFC in stabilizing the electrical power if the supercapacitor was not pre-charged. The PEMFC–SC direct hybridization system with a non-pre-charged supercapacitor consumed the highest volume of hydrogen (30.50 ml) to generate 1 mA of current, while the standalone PEMFC required only 16.90 ml of fuel. On the other hand, the PEMFC–SC (0.90 V) utilized the lowest quantity of hydrogen gas, which was 14.30 ml/1 mA. The analysis of hydrogen cost in the production of 1 mA of electricity showed the same tendency as the hydrogen volume analysis. When focusing on the cell voltage at 10 mA cm^−2^ of current density, the pre-charging supercapacitor acted as an auxiliary electric source, reducing the voltage degradation loss of the hybrid system.^23^ In terms of the fuel cost determination, the HP grade of hydrogen was utilized, and the price of 7 m^3^ (7 000 000 ml) of hydrogen was 59 283.35 USD. Thus, the price of hydrogen per milliliter was 0.0085 USD. In brief, the use of a supercapacitor to solve the energy management problem and enhance the fuel economy is obviously promising (Table 1); however, pre-charging the supercapacitor is necessary. The optimum charging voltage must be determined in future work.

**Table 1:** Results of the fuel economy analyses of the PEMFC–SC direct hybridization system^a^

System	Volume of H_2_ for generating 1 mA (ml)	Cost of H_2_ for generating 1 mA (USD)	Generated *V* at a current density of 10 mA cm^−2^
PEMFC	16.90	0.1437	0.70
PEMFC–SC (0.90 V)	14.30	0.1216	0.80
PEMFC–SC (0.45 V)	23.90	0.2032	0.30
PEMFC–SC (0.00 V)	30.50	0.2593	0

a 31.66 USD = 1.00 THB (exchange rate).

#### Comparison of the system performance of the PEMFC and PEMFC–SC direct hybridization systems

The performance of PEMFC and PEMFC–SC direct hybridization systems, which is discussed in this section, comprises their current generation, voltage losses, power generation, energy generation, yield generation, efficiency, and durability. Fig. 7 shows a performance comparison between the PEMFC and PEMFC–SC direct hybridization systems. The results show that the PEMFC–SC direct hybridization system provided higher limiting current density than the PEMFC system. This is because the supercapacitor possesses a certain amount of energy from the pre-charging process, which is integrated with the supported energy from the PEMFC, resulting in energy balance in the system. The synergistic effect of these integrated power sources enabled the system to generate the desired power density to meet the load demand. As can be seen from the polarization curves, the voltage loss of the PEMFC–SC direct hybridization system was significantly lower than the loss of the PEMFC system. It is worth noting that all losses, including activation loss, ohmic loss, and concentration loss, demonstrated the same trend in the differentiation. Activation loss is caused by slowdown of the reaction process taking place on the electrode surface. The voltage decreases because the energy in the fuel cells is utilized to drive an electrochemical reaction related to the reaction kinetics. The energy transferred from the supercapacitor to the PEMFC can help the cell to respond to the required load demand and to reduce the likelihood of creating degradation of materials. The ohmic loss is caused by resistance to the flow of ions in the electrolyte and electrons through the cell hardware and various interconnections, while the concentration loss occurs when there is a decrease in reactant concentration at the surface of the electrodes. The losses of electrons and ions during transfer result in losses of cell voltage at a certain produced current. The energy from the supercapacitor can compensate for the energy consumed by the restraint. Furthermore, the efficiency exhibited 53% improvement in the PEMFC–SC direct hybridization system and 36% improvement in the PEMFC system.

The energy densities of the PEMFC–SC direct hybridization and PEMFC systems, calculated at BOL, were 1.37 and 0.28 mW h, while the energy yields (the energy production per a certain amount of fuel) were 0.022 and 0.005 mW h ml^−1^, respectively. The calculated results in Table 2 illustrate that the energy and yield produced by the PEMFC–SC direct hybridization system depended upon the operating duration. If the hybrid system was operated at a current density of 15.625 mA cm^−2^ in each stage of the stress test profile (Fig. 4), both values decreased with increasing operating time. This is because voltage degradation was caused by material degradation, so the hybrid system could not produce stable electrical voltage at the same current density.

**Table 2:** The energy and yield of the PEMFC–SC direct hybridization system

Parameters	BOL	Conditioning after BOL	Load cycling	Conditioning after load cycling	Start–stop cycling
Energy (mW h)	3.28	2.45	2.34	2.19	1.88
Yield (mW h ml^−1^)	0.0164	0.0123	0.0117	0.0110	0.0094

**Fig. 8:**
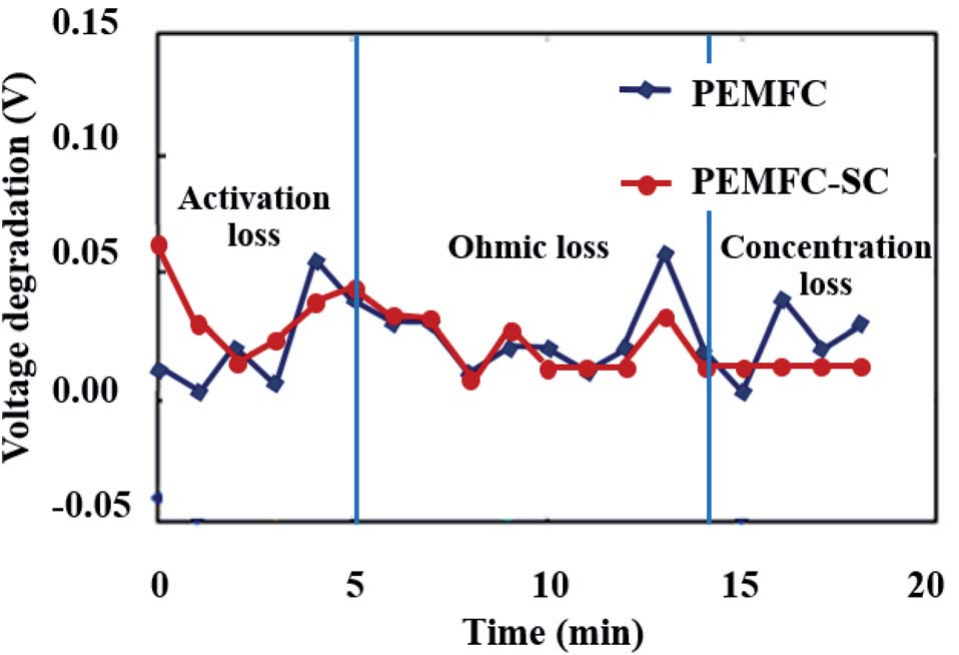
Voltage degradation of the PEMFC and PEMFC–SC direct hybridization systems.

Fig. 8 illustrates the durability of the two systems. The graph of cell voltage *versus* time shows that the high fluctuation of the PEMFC caused by electrical energy in the PEMFC was not clear. If the PEMFC supplied energy to load the voltage, the degradation dramatically increased. All the data point to the positive effects of pre-charging the supercapacitor on the performance of PEMFC–SC hybridization systems. The characteristics of voltage degradation as a function of time can be interpreted from three zones of current density which indicated major losses: activation loss, ohmic loss, and concentration loss. The activation loss located in the range of 0–5 mA cm^−2^ showed the highest voltage degradation because the energy was consumed to drive an electrochemical reaction. If electrons transfer through an electrical medium, heat will be generated. The produced heat transforms into kinetic energy that is utilized to drive a redox reaction. The activation loss of the PEMFC–SC direct hybridization system was higher than the loss of the PEMFC system because of the fast discharging process of the supercapacitor. The tendencies of voltage degradation of these two systems in the ohmic loss region (5–16 mA cm^−2^) were similar. The voltage loss resulted from the resistance of electrons and charges. In the concentration loss zone (16–23 mA cm^−2^), the PEMFC–SC direct hybridization system provided better performance than the PEMFC system. The supercapacitor acted as a second power source to supply current to the load, leading to a decrease in fuel and oxidant starvation.

### PEMFC–SC direct hybridization system characterization using EIS

#### The parameter selection for the EIS investigation

**Fig. 9:**
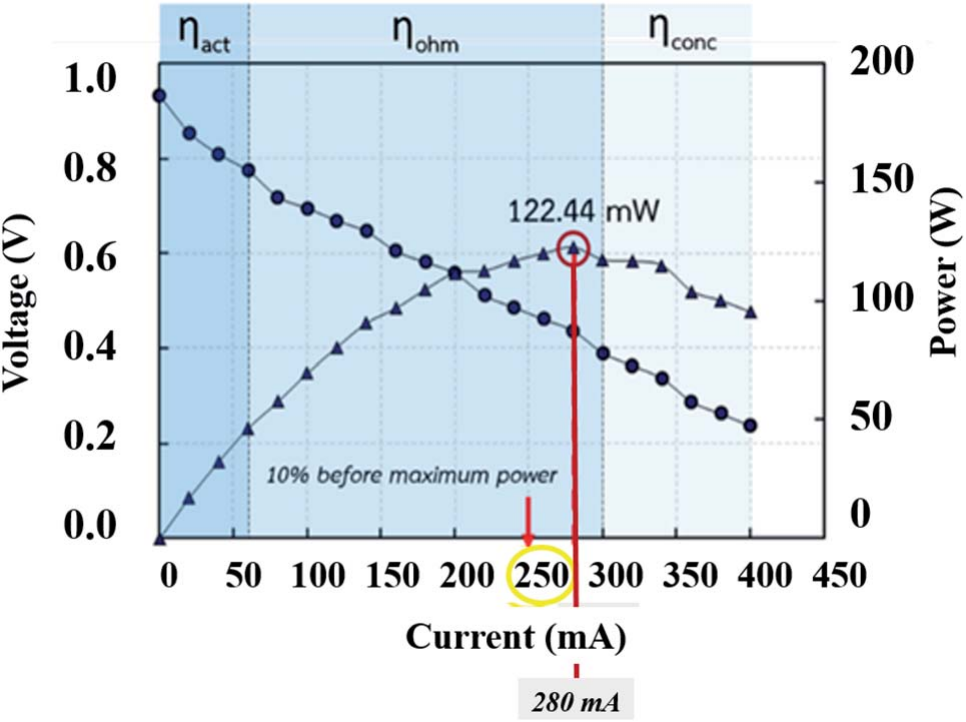
Current selection from the polarization curve for the EIS procedure.

An important parameter for an EIS procedure is the current supplied from the function generator (AC source). The current setting was selected as 10% before the maximum power density located in the ohmic loss zone (Fig. 9). The other setting parameters were swept frequency from 0.1 to 5000 Hz, 63.0 mV_pp_ of amplitude, 346.5 mV of HiLeV, 283.0 mV of LoLeV, and 315.0 mV DC of offset. The information was fitted to a suitable circuit model by the MATLAB program to create Nyquist plots.

#### Effects of the supercapacitor on impedance

**Fig. 10:**
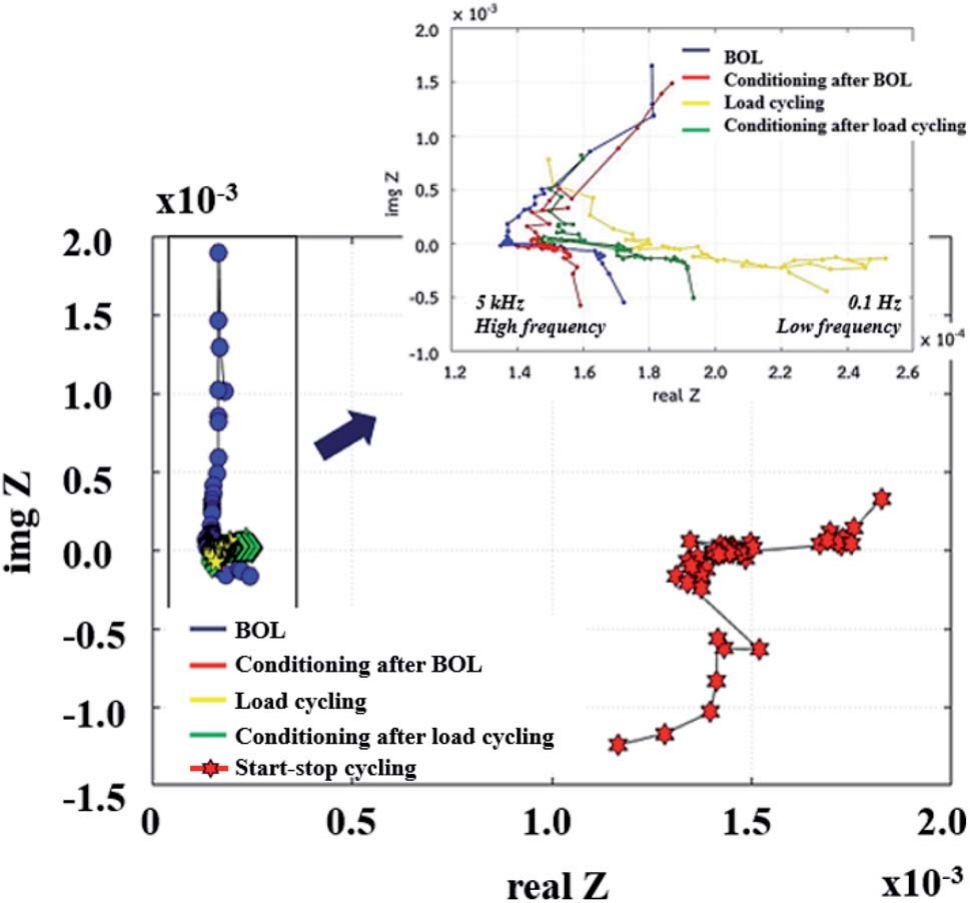
Nyquist plots of PEMFC–SC after BOL, CBOL, LC, CLC, and SS testing.

The physical and chemical mechanisms of a PEMFC are typically investigated using the EIS technique. The Nyquist plot can generally be divided into three zones, including high frequency, representing dominant resistive nature, middle frequency, showing Warburg diffusion, and low frequency, indicating capacitive behaviour.^14^ The PEMFC–SC direct hybridization system was analyzed by following the accelerated stress test procedure described in the Experimental section. The EIS diagnoses (Fig. 10) were performed after each step as follows: the beginning of life (BOL), conditioning after BOL (CBOL), load cycling (LC), conditioning after LC (CLC), and start–stop cycling (SS).

**Fig. 11:**
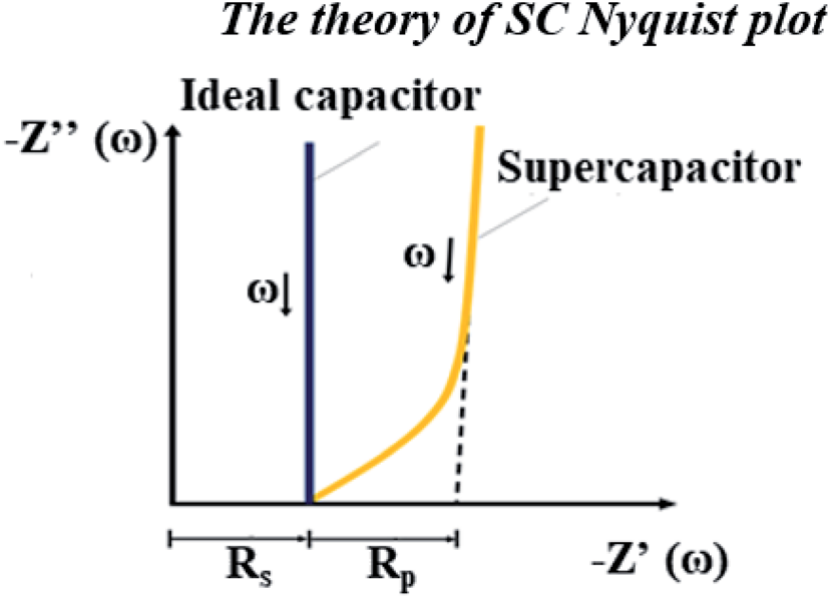
Nyquist plot of an ideal capacitor.^14^

Unfortunately, this integrated system was difficult to diagnose by EIS due to the behavior of the supercapacitor, which interrupted the PEMFC impedance. Because these were integrated systems, the Nyquist plot of an ideal supercapacitor was used as a reference to ensure that the plots were reasonable. In the case of an ideal supercapacitor (Fig. 11), the shift of the vertical line in the *x*-axis direction identifies the equivalent series resistance (ESR) of the device under test, and the vertical slope exhibits a capacitance that is invariant with frequency. The plot of the individual supercapacitor shows two distinct regions. One is the region where the graph has a 45° slope, which is being encountered at higher frequencies. This portion of the graph corresponds to the diffusion process of the ability of ions to penetrate the pores. The resistance from the EIS measurement is typically modeled by a distributed resistance *R*_p_ and a distributed capacitance. The Nyquist plot of PEMFC–SC-BOL can be seen more distinctly than the other plots because it looks similar to the plot of the ideal supercapacitor. Its behavior at low frequency (the straight line vertical to the *x*-axis) proposes capacitive behavior of ionic and electronic charges in the double layer of the supercapacitor electrode.^24–26^

The supercapacitor during PEMFC–SC-BOL testing had a higher capacity than during PEMFC–SC-CBOL, PEMFC–SC-LC, and PEMFC–SC-CLC testing because the supercapacitor stored high charges to provide a higher voltage than in the other profiles. This hypothesis can be supported by the polarization curve, which provides the highest power (296.81 mW). The performance of the system in the other steps, PEMFC–SC-CBOL, PEMFC–SC-LC, PEMFC–SC-CLC, and PEMFC–SC-SS, is 218.05, 200.14, 185.23, and 102.48 mW, respectively. The Nyquist plot measured after start–stop cycling appeared in the low-frequency zone because the PEMFC gained higher impedance or higher resistance. This provides evidence of the strong influence of humidity cycling and accelerated stress conditions on the degradation of the materials.

### PEMFC–SC working behavior based on the accelerated stress test profile

**Fig. 12:**
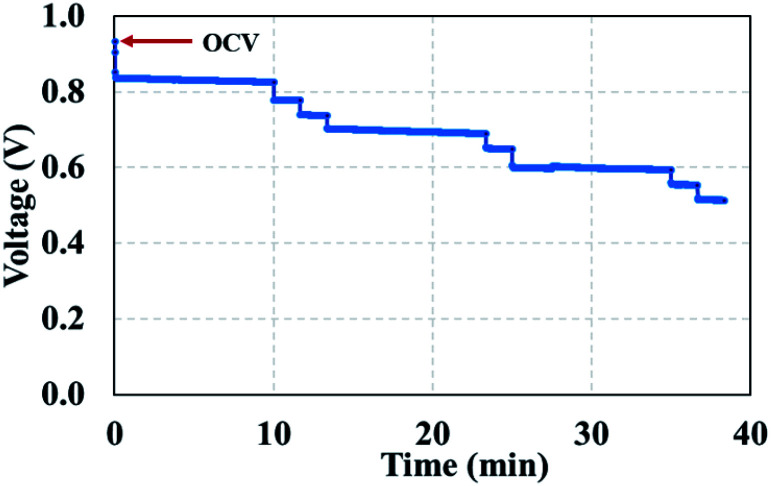
Voltage profile of the BOL testing.

The voltage of the PEMFC–SC hybridization system can be automatically managed by the base voltage level of a supercapacitor. The working behavior of this system under the stress test profiles was uncertain, similar to the voltage behavior of the system at the beginning of life (Fig. 12). The open circuit voltage (OCV) equals 0.92 V at BOL, and then the voltage gradually drops, corresponding to the created stress test profile (Fig. 4). The stress profile was designed to increase the generated current in quasi-static steps; therefore, the system voltage decreased in steps.

**Fig. 13:**
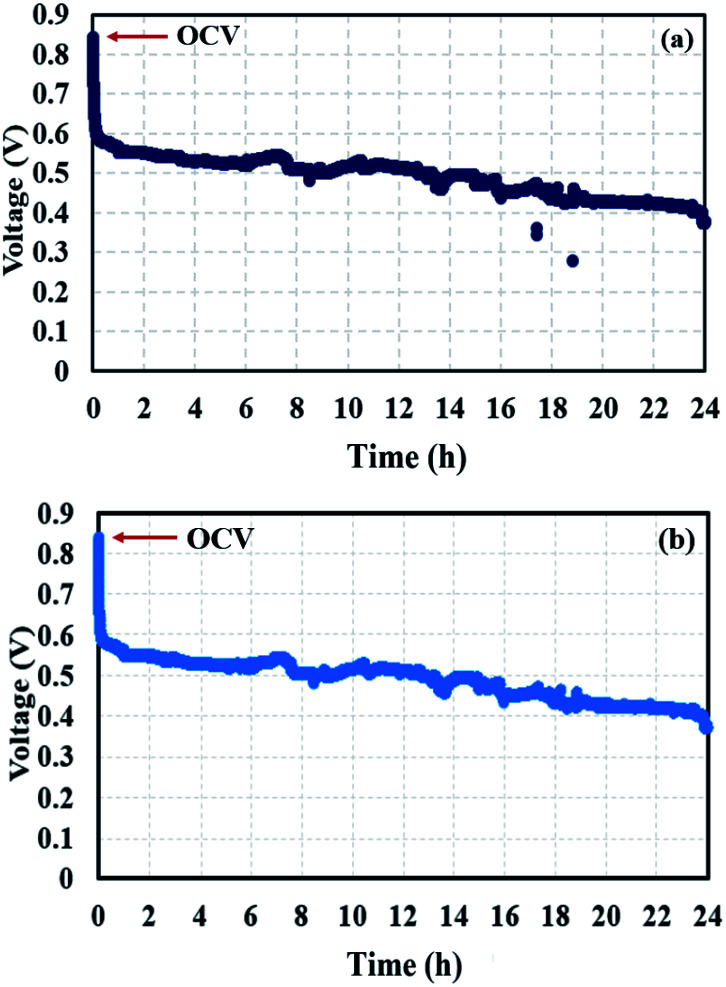
Voltage profiles of conditioning (a) after BOL testing and (b) after quasi-static load cycling conditions.

The voltage profiles at the conditioning stages (Fig. 13a and b) reveal a decrease in voltage due flooding phenomena arising in the cell. Water impeded the active sites of the catalyst; therefore, the reactants could not reach those active sites, resulting in an incomplete redox reaction. EIS measurements were taken to support the theory of flooding occurrence.

**Fig. 14:**
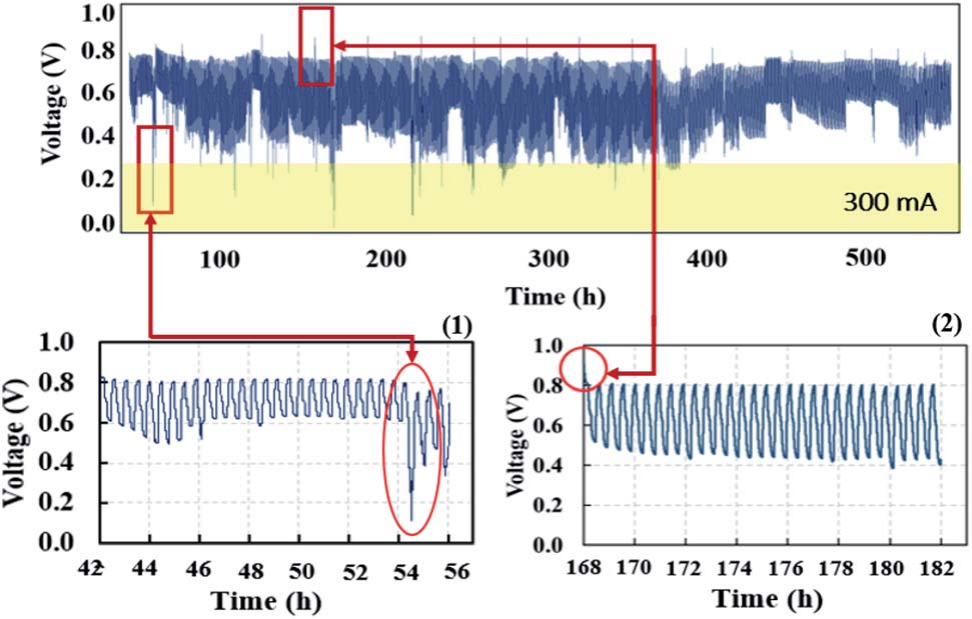
Voltage profiles of quasi-static load cycling condition testing.

**Fig. 15:**
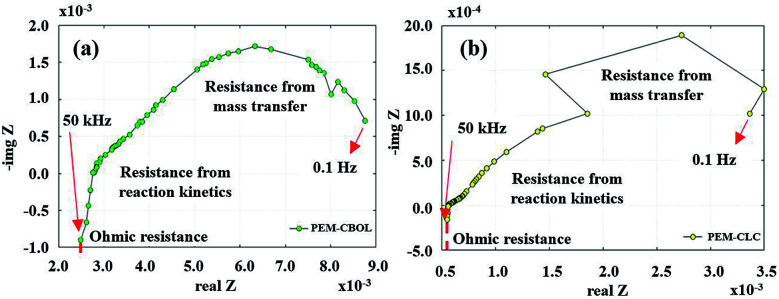
Nyquist plots from conditioning after (a) BOL testing and (b) quasi-static load cycling.

The voltage profile in quasi-static load cycling (Fig. 14) can be divided into two characteristics. First, the voltage spikes in the range of 0.7–0.8 V occur from the fast discharging of the supercapacitor. During the cycling, there were peak loads in repetitions that required fast responses; therefore, the supercapacitor suddenly provided voltage to meet the load demand, leading to spikes in cell voltage. Second, the voltage dropped in the range of 0.1–0.3 V, which was caused by a high current demand at 300 mA. To run the hybrid system long term with high current demand, the generated voltage from the PEMFC must be charged to the supercapacitor, which is affected by a drop in voltage. During this step, the PEMFC–SC hybridization system experienced voltage degradation because the voltage could be steadily provided to meet the load demand. The PEMFC–SC hybridization system was operated through quasi-static load cycling and start–stop cycling to accelerate cell degradation relating to actual application. An EIS technique was applied to diagnose the characteristics of cell degradation occurring after the PEMFC–SC hybridization system was operated through both cycling tests. Note that the Nyquist plots in Fig. 15 and 17 were obtained from testing a fuel cell disconnected from a supercapacitor to prevent disturbance from the supercapacitor behavior. The Nyquist plots in Fig. 15a and b show that more transport effects existed after quasi-static load cycling than in the circumstances after BOL. The semicircular region in Fig. 15b appears to consist of a tilted spike in the low-frequency region.

**Fig. 16:**
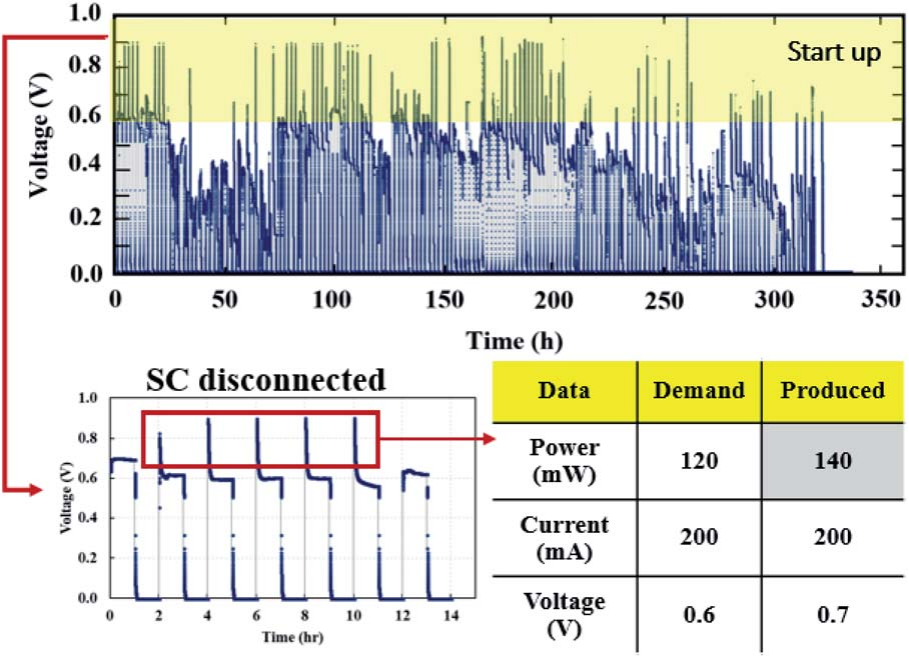
Voltage profile of start–stop condition testing.

The cell voltage profile from the start–stop conditions (Fig. 16) illustrated the same short-term electrical transients in voltage that occurred in the load cycling profile. The PEMFC–SC hybridization operated for 504 hours under quasi-static load cycling, whereas it responded to load demand under start–stop cycling conditions for only 336 hours before irreversible degradation started to occur.

**Fig. 17:**
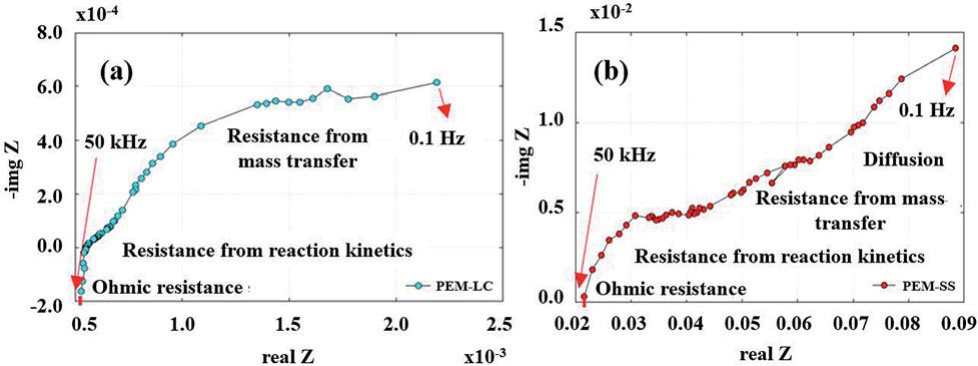
Nyquist plots from (a) quasi-static load cycling testing and (b) start–stop testing.

The EIS results (Fig. 17a) show an irregular semicircle in the Nyquist plot, which implies a relatively slow charge transfer process at higher frequencies. Furthermore, the diagonal “Warburg” responded in a low-frequency zone. These changes in the Nyquist plot point to material degradation, such as pinholes in the membrane and crossover of the reactant to the other side. The Nyquist plot in Fig. 17b presents the same characteristics as in the quasi-static load cycling but with greater impact. The catalyst degradation may be generated by Warburg diffusion because the reactant diffused onto the catalyst layer and into the porous catalyst support. These phenomena cause corrosion of the carbon support and/or platinum dissolution, corresponding to eqn (10)–(13).

Corrosion of carbon support^2^

10 C + H_2_O → CO_2_ + 4H^+^ + 4e^−^, *E*_cell_ = 0.207 V

Platinum dissolution^2^

11 Pt → Pt^2+^ + 2e^−^, *E*_cell_ = 1.188 V

12 Pt + H_2_O → PtO + 2H^+^ + 2e^−^, *E*_cell_ = 0.980 V

13 PtO + 2H^+^ → Pt^2+^ + H_2_O + 2e^−^, *E*_cell_ = 0.208 V

**Fig. 18:**
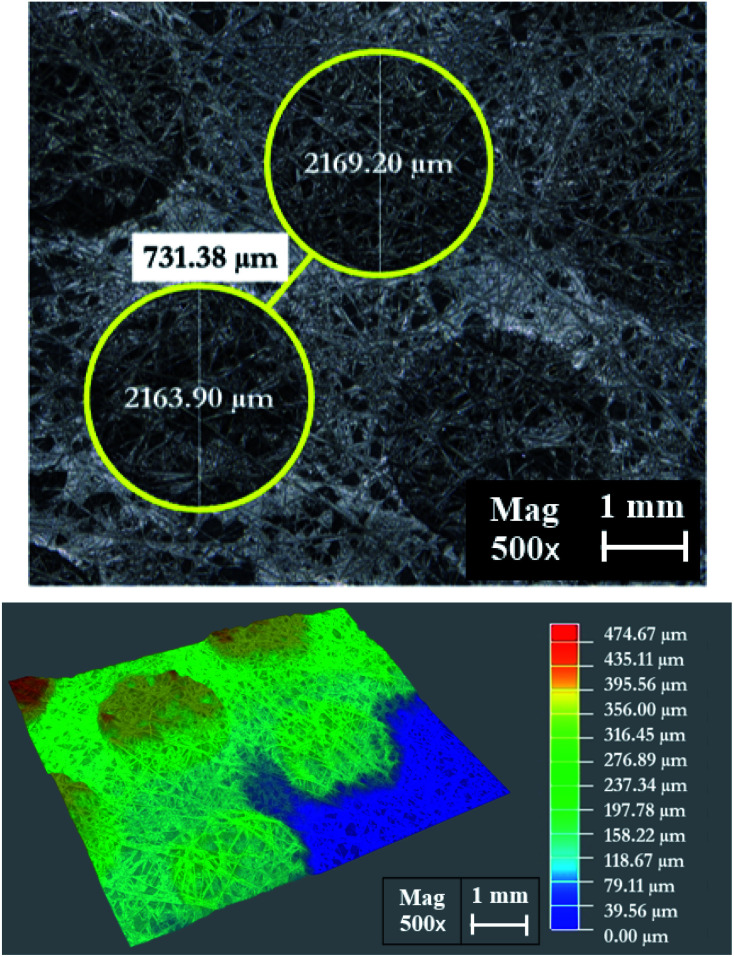
The MEA surface area morphology as observed by a Leika DM4 M microscope.

**Fig. 19:**
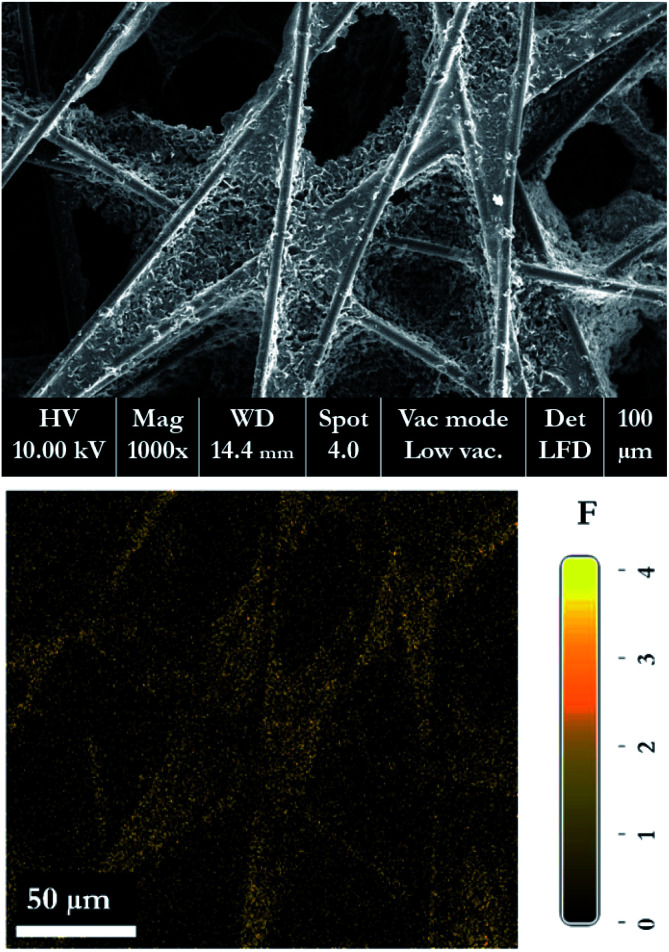
SEM-EDX micrograph of the GDL, showing the fluorine released from the membrane structure to the GDL layer.

Fig. 18 shows the morphology of the membrane electrode assembly (MEA) after the accelerated stress test was finished. The MEA was mechanically stressed under compressive pressure; this may cause cracking, membrane thinning, pinhole creation, and catalyst delamination in the gas diffusion layer (GDL) and/or MEA. The failure of the membrane from chemical degradation may have occurred due to the scenario described in eqn (14)–(16),^27^ where the surface of the GDL, which is made from carbon fiber, is damaged by fluorine released from the membrane during the generating process (Fig. 19).

Chemical degradation of the membrane:^28^

14 R-CF_2_COOH_2_ + HO* → R-CF_2_ + CO_2_ + H_2_O

15
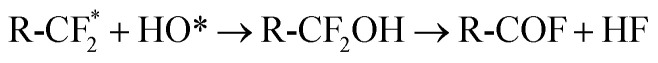

16 R-COFH + H_2_O → R-COOH + HF

## 4 Conclusions

This article deals with the observations of the performance of a PEMFC–SC direct hybridization system following an accelerated stress test in terms of voltage degradation, energy management, energy balance, and degradation of materials. Electrochemical impedance spectroscopy and scanning electron microscopy techniques were employed because they are very suitable and useful methods. The PEMFC–SC direct hybridization system is confirmed to be useful for dynamic operating conditions based on driving behaviors. The PEMFC acts as the main source of energy due to its ability to provide and meet high energy density, while the supercapacitor functions as an auxiliary source. Even though the supercapacitor possesses abilities, such as fast charging and discharging, that can provide power to meet dynamic load quickly, it requires a pre-charging process to balance the voltage levels. The impact of the charging voltage on hydrogen consumption was observed to evaluate the capability of the pre-charging process. However, the optimization of the pre-charging procedure should be further studied in depth to determine suitable parameters such as charging voltage, time and the effects of temperature variations for efficient energy storage. The PEMFC–SC direct hybridization combination shows superior performance to the standalone PEMFC. It generated a maximum voltage of 0.90 V, 21.88 mA cm^−2^ (350 mA) maximum current density, 10.20 mW cm^−2^ maximum power density, 1.37 mW h maximum energy density, 0.022 mW h ml^−1^ maximum yield, and 53% efficiency of the system. The experimental results of the final validation test of the accelerated stress test procedure are presented. Consistent voltage degradations of quasi-static load cycling and start–stop cycling were investigated. The start–stop cycling in the time and energy domain indicates more influence than the quasi-static load cycling because it induces relative humidity cycle, temperature cycle, and starvation, conveying the highest ohmic resistance and directly affecting the degradation of the material in the PEMFC–SC direct hybridization system. As can be seen, the PEMFC–SC direct hybrid system can be operated for only 336 hours instead of 504 hours, as predicted, because of irreversible degradation occurring in the system. The test results show the possibility of water flooding, GDL and/or MEA cracking, membrane thinning, pinhole creation, catalyst delamination, corrosion of carbon support and/or platinum dissolution in the system after running for a long period of time. To understand more about these phenomena related to fluctuating load demand and energy management, advanced physicochemical and more electrochemical characterizations must be applied for further studies. The authors of this work believe this project to be a good guideline for the modification of integrated alternative energy resources and observed feasibility of real-life applications in terms of performance, reliability, and durability.

## Conflicts of interest

There are no conflicts to declare.

## Supplementary Material
